# Spontaneous hepatic rupture: A catastrophic complication in a patient with primary splenic angiosarcoma and hepatic metastasis: case report and literature review

**DOI:** 10.3389/fonc.2025.1708613

**Published:** 2025-11-18

**Authors:** Zhidan Wu, Xiangming Cao, Yuanyuan Zuo, Yongqiang Zheng

**Affiliations:** 1 The Department of Laboratory, Jiangyin People’s Hospital, Jiangyin, Jiangsu, China; 2 The Department of Oncology, Jiangyin People’s Hospital, Jiangyin, Jiangsu, China; 3 The Department of Pathology, Jiangyin People’s Hospital, Jiangyin, Jiangsu, China; 4 The Department of Radiology, Jiangyin People’s Hospital, Jiangyin, Jiangsu, China

**Keywords:** primary splenic angiosarcoma, liver metastasis, splenic rupture, liver rupture, case report, sunitinib

## Abstract

**Background:**

Primary splenic angiosarcoma (PSA) is an exceedingly rare and aggressive malignancy with a poor prognosis. This report aims to present a rare case of PSA that progressed to fatal hepatic rupture due to rapid metastatic spread following splenectomy and adjuvant therapy; a review of the relevant literature is also provided to discuss the diagnostic and therapeutic challenges associated with this condition.

**Case presentation:**

A 52-year-old male patient presented with acute abdominal pain and hypovolemic shock. Imaging revealed splenic rupture with hemoperitoneum. An emergency splenectomy was performed, and histopathological examination confirmed the diagnosis of angiosarcoma. The patient received postoperative chemotherapy (liposomal paclitaxel) and subsequent targeted therapy (sunitinib). However, rapid hepatic metastasis occurred, leading to spontaneous hepatic rupture and death 6 months after the initial diagnosis.

**Conclusion:**

The condition poses a diagnostic and therapeutic challenge due to its nonspecific presentation and high aggressiveness. Early splenectomy remains the prevailing standard of care. This case demonstrates the potential inefficacy of sunitinib against PSA-derived hepatic metastases and emphasizes the critical importance of early diagnosis and intervention before splenic rupture occurs. The potential for combination therapies, including immunotherapy, to represent future investigative avenues is a promising area for future research.

## Introduction

Primary splenic angiosarcoma (PSA) is an exceptionally rare and aggressive malignant vascular tumor that originates from splenic vascular endothelial cells, particularly those of mesenchymal origin within the splenic sinus network. Its estimated incidence ranges from 0.14 to 0.23 cases per million, with the condition predominantly affecting individuals between the ages of 50 and 79, exhibiting a reported male-to-female ratio of approximately 4:3 (1, 2). Since its initial description by Langhans in 1879, the documented cases in English literature have numbered fewer than 300 (3–5).

This study explores the clinical characteristics of PSA, emphasizing its highly aggressive behavior and frequent diagnosis at advanced stages. We highlight the necessity for enhanced clinical awareness and the development of improved management strategies. Existing literature consists largely of isolated case reports, and there is a paucity of comprehensive clinical guidelines. This case report describes a patient with PSA who presented with spontaneous splenic rupture. Despite undergoing 4.5 cycles of paclitaxel-based chemotherapy and 1 month of sunitinib targeted therapy, the patient ultimately succumbed to acute liver rupture resulting from rapidly progressive metastatic dissemination. The rapid development of hepatic metastasis after splenic rupture, coupled with the lack of response to sunitinib, offers critical insights into the aggressive behavior of PSA and underscores the need for more effective treatment approaches.

## Case report

### Initial presentation and emergency intervention (August 2023)

**Figure 1:**
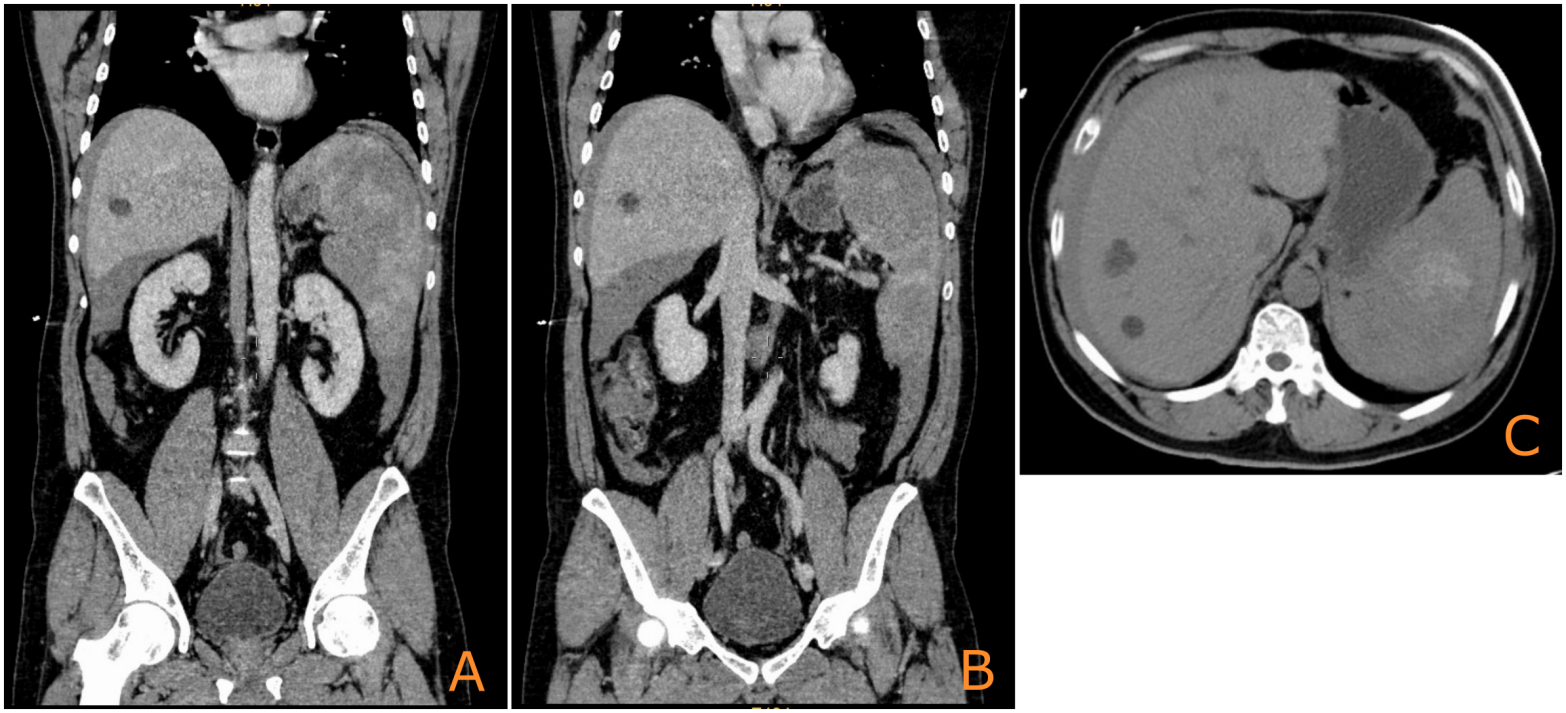
Initial CT image of the spleen upon admission showing multiple nodular occupations [**(A, B)**: coronal plane; **(C)**: axial plane].

A 52-year-old man was admitted on 8 August 2023, with acute left-sided abdominal pain and syncope without any obvious triggers 7 h earlier. The patient presented with hypotensive shock and was managed with blood transfusions and fluid resuscitation. Computed tomography (CT) imaging revealed multiple splenic lesions, substantial abdominal and pelvic blood accumulation, and multiple cysts in the liver (**Figure 1**). The results of the laboratory tests indicated the presence of severe hemorrhage (Hb: 90 g/L) and critical thrombocytopenia (PLT: 23×10^9^/L). Concurrently, the serum levels of the tumor markers CEA, CA125, CA153, CA199, and CA242 were all found to be within normal ranges. In view of the hemodynamic instability, which was indicative of ongoing hemorrhage, an emergency splenectomy was performed.

### Surgical findings and pathological diagnosis

**Figure 2:**
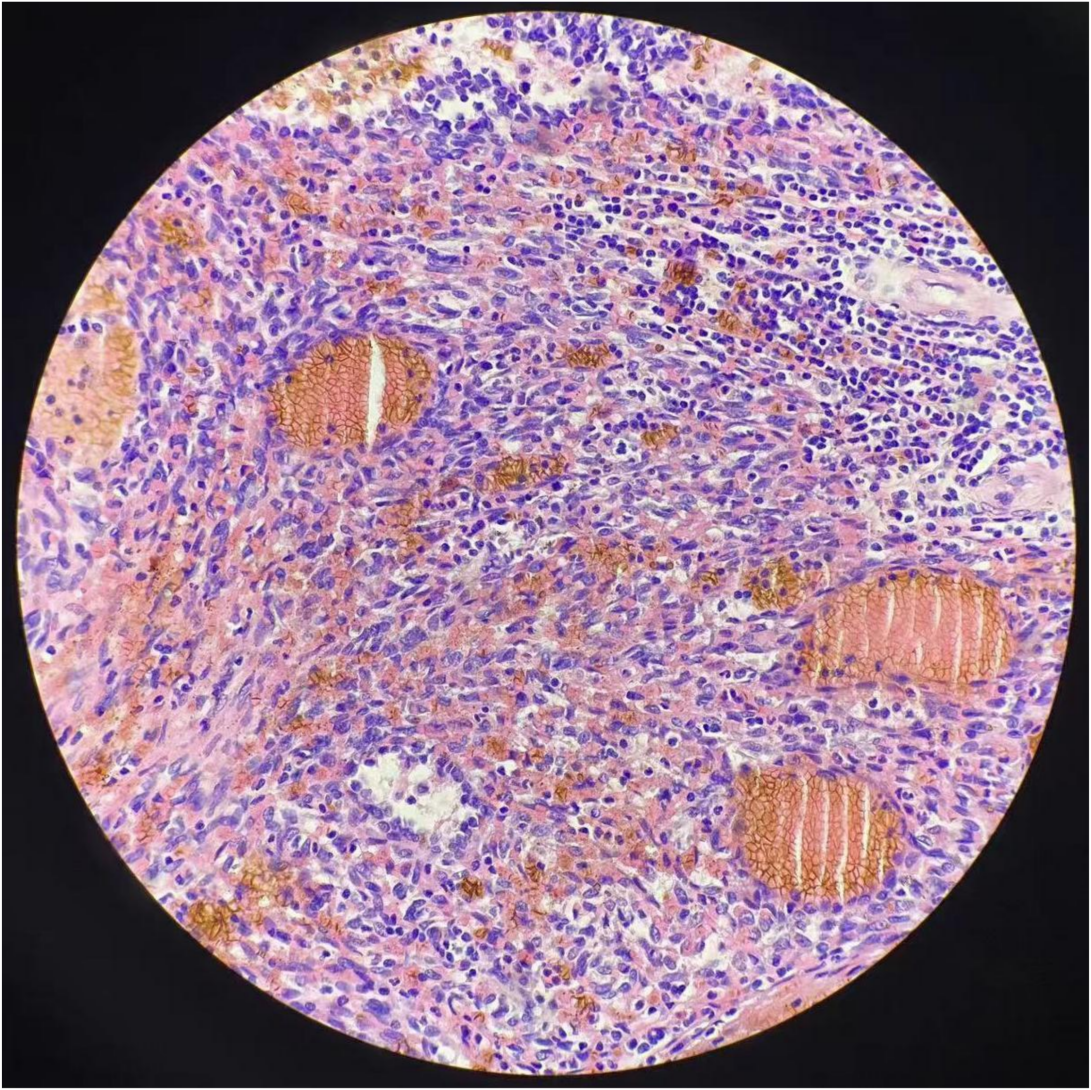
Hematoxylin and eosin (H&E) staining reveals regular nodules consisted of hyperplastic, dilated blood vessels with lumens of varying sizes, some with hemorrhage and necrosis.

**Figure 3:**
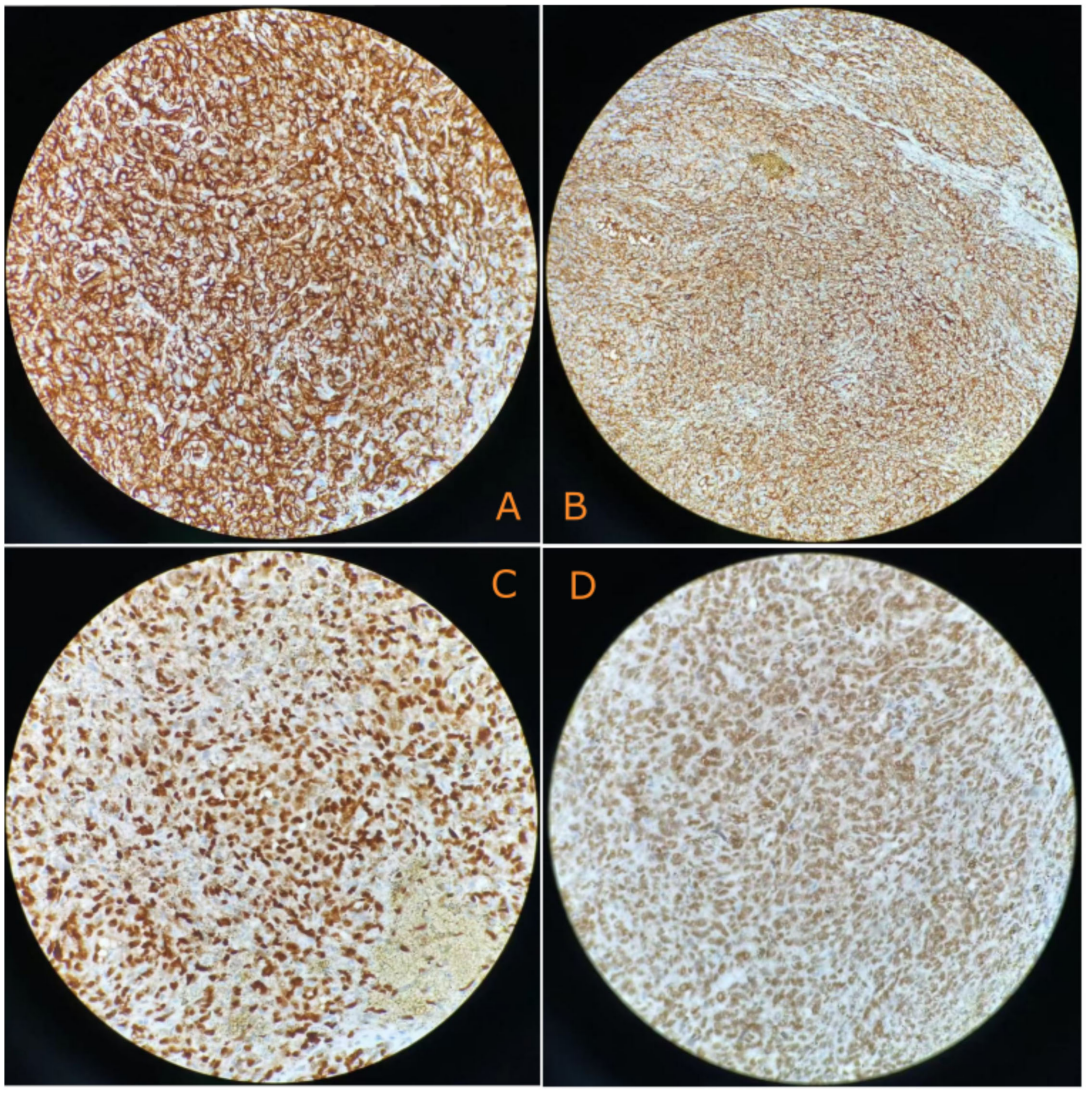
Immunohistochemical characterization reveals the following: **(A)** Tumor cells diffusely expressed CD31(+) (400×). **(B)** Tumor cells diffusely expressed CD34(+) (400×). **(C)** Tumor cells diffusely expressed EGR(+) (400×). **(D)** Tumor cells diffusely expressed INI-1(+) (400×).

The surgery revealed approximately 2,000 mL of hemoperitoneum. The spleen was observed to be enlarged, with a nodular, dark red-gray cut surface and two rupture sites. Its length was measured to be approximately 6 cm. A subsequent histopathological examination of the resected spleen revealed a malignant vascular tumor (**Figure 2**). Immunohistochemical analysis showed positive staining for CD31, CD34, and ERG, a high Ki-67 proliferation index (>60%), and retained INI-1 expression, supporting a diagnosis of angiosarcoma (**Figure 3**).

### Postoperative course and adjuvant therapy

The patient recovered well and was discharged after 10 days. Subsequently, he commenced adjuvant chemotherapy with liposomal paclitaxel (150 mg, d1, d8) in late September 2023 and was completing 3.5 cycles. A surveillance CT on 27 November 2023 showed no evidence of liver metastasis. Throughout the entire chemotherapy process, the patient’s platelet count remained normal. The patient reported mild fatigue but no other discomfort and therefore continued working.

### Disease progression and outcome

A follow-up CT on 14 January 2024 revealed new multiple liver metastases. After reviewing the literature (6), the clinician initiated an experimental targeted therapy based on sunitinib (50 mg daily). Despite this, the patient presented with acute upper abdominal pain on 10 February 2024. CT confirmed hepatic rupture with hemorrhage. The patient opted for conservative management and succumbed shortly afterwards.

### A timeline with relevant data

The patient’s clinical course, detailed in **Table 1**, was marked by the following: subsequent to the initial discovery of a substantial splenic mass, the patient did not accord it sufficient attention and did not undergo any subsequent follow-up examinations during the ensuing year. One year later, the patient was readmitted to hospital urgently due to spontaneous rupture of the spleen. Despite undergoing a splenectomy, followed by postoperative chemotherapy and targeted therapy, the patient’s survival was limited to a mere 6 months.

**Table 1:** Timeline of diagnosis, treatment, and supporting investigations for patients with liver metastases following PSA surgery.

Hospital day	Clinical events and interventions	Imaging findings	Key laboratory findings
21 August 2022	Admission for acute abdominal pain. No intervention measures were taken.	MRI: A large mass in the spleen.	–
8 August 2023	Admission for acute left-sided abdominal pain and syncopy.	CT: Splenic rupture.	Hb: 90 g/L, PLT: 23×10^9^/L
9 August 2023	Hypotensive shock. Total splenectomy.	–	Hb: 80 g/L, PLT: 22×10^9^/L
29 September 2023	Adjuvant chemotherapy with liposomal paclitaxel (150 mg, d1, d8).	CT (29 Sep.): No evidence of liver metastasis.	Hb: 128 g/L, PLT: 171×10^9^/L (9 Oct.)
14 January 2024	Add oral sunitinib 50 mg daily as targeted therapy.	CT: Revealed new multiple liver metastases.	–
10 February 2024	Admission for acute right upper abdominal pain.	CT: Hepatic rupture with hemorrhage.	Hb: 87 g/L, PLT: 39×10^9^/L

## Discussion

### Diagnostic challenges and pathological features

PSA is notoriously difficult to diagnose preoperatively. The definitive diagnosis of PSA depends on biopsy or pathological examination. However, splenic biopsy is considered for use with caution owing to the high risks of procedure-induced rupture and metastasis, as well as its limited diagnostic utility (7). As seen in our case and others, spontaneous rupture is often the presenting symptom. Hemorrhage represents the primary laboratory abnormality in PSA, accompanied by other findings such as leukopenia, thrombocytopenia, and an elevated erythrocyte sedimentation rate (8). The definitive diagnosis relies on pathology and IHC, as demonstrated by the positive staining of vascular markers observed in our patient.

### Treatment strategies and literature comparison

Splenectomy is the primary and potentially curative treatment, especially if performed prior to rupture or metastasis (9). The role of adjuvant therapy remains poorly defined, as most chemotherapeutic regimens are empirically derived and lack support from large-scale clinical evidence. According to reports, treatment regimens based on paclitaxel or anthracycline chemotherapy are commonly used (10, 11). A retrospective analysis of 28 patients with soft tissue sarcoma demonstrated a clinical benefit rate of 50% in evaluable patients receiving at least four cycles of nivolumab combined with pazopanib (12). In a multicenter phase II trial, sorafenib exhibited activity in metastatic angiosarcoma, with tumor recurrence following treatment cessation—a pattern consistent with other targeted therapies in oncology (13). Another open-label, multicenter phase II study indicated that bevacizumab has promising efficacy in angiosarcoma, achieving stable disease in 11 patients with a favorable safety profile (14). In 2022, Pan et al. reported a case of hepatic metastasis from PSA that was managed with splenectomy followed by adjuvant therapy combining sorafenib and camrelizumab, resulting in no recurrence or metastasis after 15 months of follow-up (15). These reports suggest potential for targeted and immunotherapy combinations.

### Efficacy of sunitinib and aggressive tumor behavior

Our case contributes to the limited experience with sunitinib in PSA. The rapid disease progression culminating in hepatic rupture soon after starting sunitinib suggests either innate or rapidly acquired resistance. This resistance is likely multifactorial in nature, primarily driven by the tumor’s exceptionally high proliferative capacity, as evidenced by the Ki-67 index exceeding 60% (8). Such an immense proliferative drive may simply overwhelm the primarily cytostatic effect of a single-agent tyrosine kinase inhibitor (TKI). Moreover, highly aggressive angiosarcomas exhibit considerable genomic plasticity, allowing them to activate alternative signaling pathways (e.g., MET, AXL, and MAS) (13, 16), thereby bypassing VEGFR inhibition by sunitinib. Concurrently, sunitinib may have inadvertently exacerbated the structural abnormality and fragility of the tumor vasculature within the metastases, promoting a hypoxic, acidic, and pro-invasive tumor microenvironment (17, 18).

### Lessons on early intervention

Tumor rupture leads to the dissemination of a large number of highly invasive and proliferative tumor cells into the peritoneal cavity. Once infiltrated by substantial tumor cells, the peritoneal cavity becomes a reservoir for subsequent implantation and growth of tumors, particularly in the highly vascularized liver. This offers a plausible explanation for the rapid and extensive liver metastases observed shortly after surgery. Although splenectomy is a cornerstone of radical therapy for PSA, its therapeutic efficacy is significantly compromised when performed under emergency conditions caused by tumor rupture.

**Figure 4:**
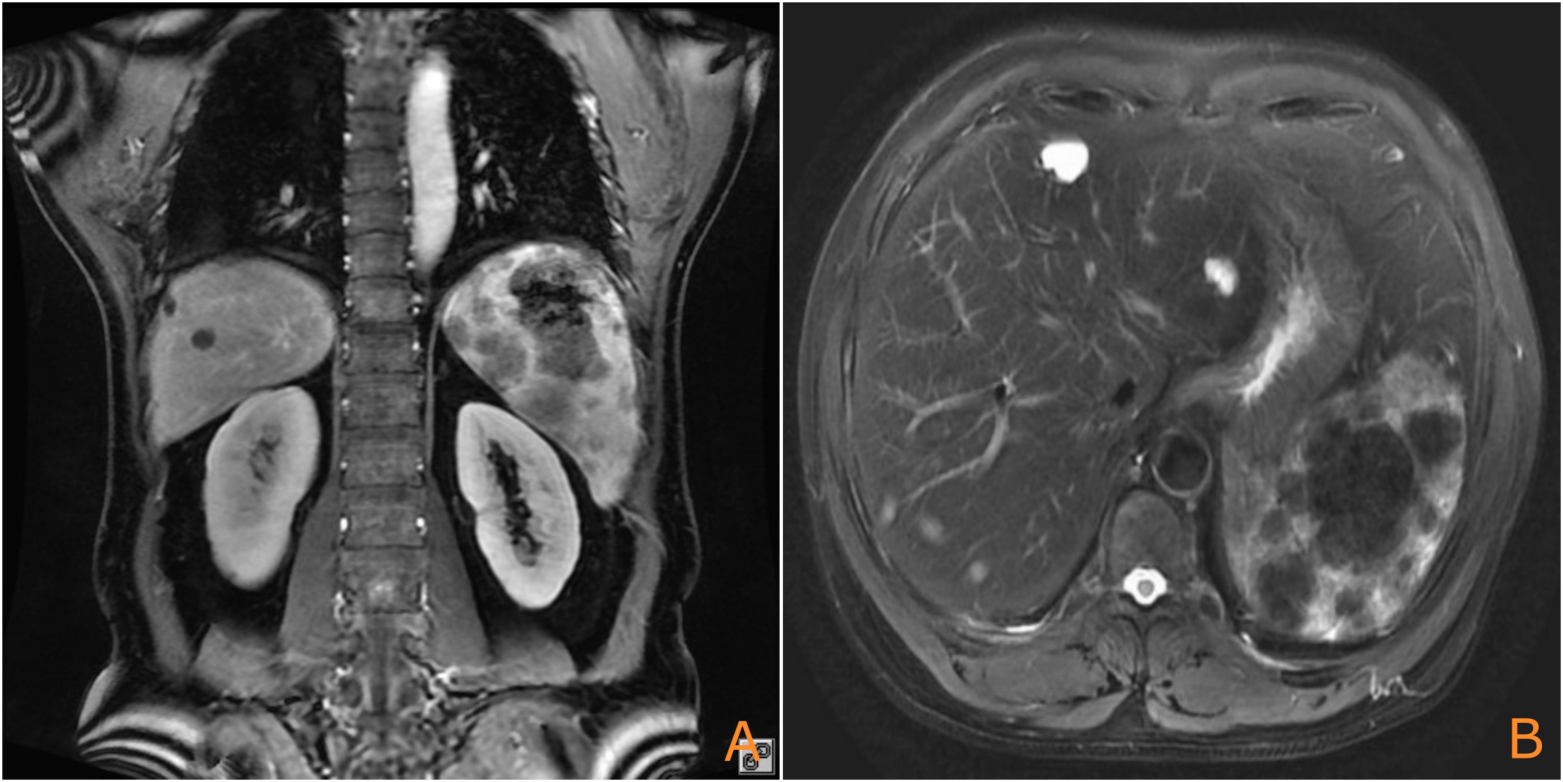
Prior MRI (August 2022) showing the splenic mass (arrow), which was not intervened upon [**(A)**: coronal plane; **(B)**: axial plane].

Analysis of relevant cases (**Table 2**) indicated that patients with PSA with liver metastasis who underwent splenectomy prior to rupture exhibited improved survival, with the longest observed survival reaching 48 months. This case highlights a significant missed opportunity for early intervention. One year ago (August 2022), a magnetic resonance imaging (MRI) scan performed for abdominal pain revealed a large mass in the spleen (**Figure 4**). The patient declined further investigation, thereby missing the chance for early intervention. Had a splenectomy been pursued at that time, the outcome might have been more favorable.

**Table 2:** Summary of reported cases of PSA with treatment details and outcomes.

Ref.	Report year	Age (years)	Gender	Splenic rupture	Surgical plan	Chemotherapy or adjuvant therapy regimen	Overall survival
(19)	2025	64	Female	None	None	Paclitaxel, yttrium-90 (90Y) radioembolization	More than 48 months
(20)	2024	64	Male	None	Splenectomy	Antitumor combination therapy	5 months
(15)	2022	49	Female	+	Splenectomy and liver tumor resection	Sorafenib and camrelizumab	More than 15 months
(21)	2022	57	Female	+	Splenectomy	Toripalimab and anlotinib	More than 8.9 months
(22)	2021	62	Female	None	None	Paclitaxel, doxorubicin, pazopanib, docetaxel, gemcitabine, and ifosfamide	23 months
(7)	2019	80	Female	+	Splenectomy	Paclitaxel and non-selective β-AR antagonists	6 months
(23)	2018	65	Female	+	Splenectomy	Paclitaxel	More than 6 months
(24)	2014	38	Male	None	None	Paclitaxel	1 month
(25)	2012	57	Female	None	Splenectomy and hepatectomy	Paclitaxel, pazopanib, doxorubicin, and a novel agent within a phase II trial	More than 48 months
(25)	2012	30	Male	None	Splenectomy	An anthracycline-based regimen within a randomized phase III trial, paclitaxel, gemcitabine, and docetaxel	8 months
(26)	2010	70	Female	None	Splenectomy	Gemcitabine HCl, docetaxel, ifosfamide, and doxorubicin	More than 8 months

## Conclusion

In conclusion, PSA is a highly aggressive tumor where early diagnosis and splenectomy offer the best chance for survival. Imaging is a crucial diagnostic tool, but pathology is definitive. For suspected splenic masses, initial contrast-enhanced MRI is recommended to inform the subsequent consideration of prophylactic splenectomy. This case illustrates that even with surgery and standard chemotherapy, the disease may still follow a fulminant course. The failure of sunitinib in this context indicates a need for more effective systemic therapies. Future efforts should focus on molecular profiling of PSA to identify actionable targets and explore novel combination regimens, including immunotherapy.

## Data Availability

The raw data supporting the conclusions of this article will be made available by the authors, without undue reservation.
